# Comparison of the gastrointestinal tract of a dual-purpose to a broiler chicken line: A qualitative and quantitative macroscopic and microscopic study

**DOI:** 10.1371/journal.pone.0204921

**Published:** 2018-10-19

**Authors:** Zaher Alshamy, Kenneth C. Richardson, Hana Hünigen, Hafez Mohamed Hafez, Johanna Plendl, Salah Al Masri

**Affiliations:** 1 Institute of Veterinary Anatomy, Department of Veterinary Medicine, Freie Universität Berlin, Berlin, Germany; 2 College of Veterinary Medicine, School of Veterinary and Life Sciences, Murdoch University, Murdoch, Australia; 3 Institute of Poultry Diseases, Department of Veterinary Medicine, Freie Universität Berlin, Berlin, Germany; Tokat Gaziosmanpasa University, TURKEY

## Abstract

The transition to using dual-purpose chickens is an alternative to killing male hatchlings of high performance egg-laying chickens. This study aimed to compare the gastrointestinal tract of a recently developed genetic line of dual purpose male chicken, Lohmann Dual (LD), with that of a broiler line, Ross 308. Eighty birds from each line were grown until they reached an average body weight 2000 g (5 weeks for Ross and 9 for LD birds). Six birds of each line were sampled weekly. Body weight (BW), normalized mass of gastrointestinal segments and relative length of intestine were determined. Histologically the villus height, epithelium height, crypt depth, mucosal enlargement factor and the tunica muscularis thickness were measured in jejunum and ileum. Data were regressed against body weight and genetic line. Jejunal enterocyte microvilli and junctional complexes length were measured. Normalized mass and relative length of the gastrointestinal segments were greater in LD birds than in Ross birds at all ages. After day 7 these decreased steadily over the lifetime of the birds in both genetic lines. The growth curves of the gastrointestinal segments of the LD birds were similar to those of the Ross birds. In birds of the same BW, LD birds had a significantly heavier gizzard, shorter intestine, higher jejunal villi, thicker ileal tunica muscularis and smaller ileal mucosal enlargement factor than were found in Ross birds. The large gizzard in LD chickens presumably increases the degree of food processing and enhances availability of nutrients in the orad part of the intestine leading to a lower nutrient concentration and a smaller absorption surface area in the ileum of the LD compared to the Ross chickens. The anatomical differences between the two lines are important criteria for further selection and should be considered in their feeding management.

## Introduction

The commercial chicken industry is based on the genetic development of highly productive breeds selected for either egg laying or meat production. Since males of laying breeds are slow growing, they are not profitable for meat production and are killed immediately after hatching [1]. This outcome has caused intense ethical debate [2]. One suggested approach to resolve this problem is to raise layer-type males to a live weight of 600 or 2000 g and market them as alternatives to quail or broiler chickens respectively. However, to reach a live weight of 2000+ g, the layer-type males require twice the quantity of feed and triple the time compared to specifically selected broiler breeds [1]. In a public survey conducted by Leenstra et al. (2011), of 5 potential alternatives to killing one-day-old chicks, “transillumination of the fresh eggs to determine the sex of the eggs and not incubate male eggs” and “using dual-purpose chickens” scored best (25% and 24%, respectively) out of the proposed alternatives” [1].

Investigations in the nineteen nineties of traditional dual-purpose breeds such as the Australorp, Bielefelder, New Hampshire and Rhode Island Red, reported that these breeds were inefficient in both meat and egg production in comparison to highly selected lines such as Ross and Lohmann Brown [3]. More recently developed dual-purpose chicken lines such as Walesby Specials, Dominant Red Barred, Novogen Dual and Lohmann Dual (LD) have a better body weight gain and feed efficiency than the traditional dual-purpose breeds [3]. Calculations based on current prices for eggs and meat indicate that the LD breed exceeds the performance of the other dual-purpose breeds, particularly in respect to meat production. The LD line has a better feed conversion efficiency compared to Walesby Special and Brown hybrids [3].

Functional anatomical and histological characteristics of the avian gastrointestinal tract are critical to their feed conversion efficiency [4]. The chicken's gastrointestinal tract consists of the glandular stomach (proventriculus), gizzard (ventriculus) and intestine (small and large). In the glandular stomach which is lined by glands secreting pepsin, hydrochloric acid and mucus, food is mixed with these digestive juices [5]. The gizzard is the second compartment of the stomach where powerful contractions crush the food [6]. In the small intestine, feed constituents are hydrolyzed into simple molecules, in particular; free small peptides, amino acids, free fatty acids, and monosaccharides. These molecules are absorbed in the duodenum and jejunoileum and transported via blood circulation to other tissues [7].

To facilitate maximal absorption of dietary components the intestinal mucosa is highly convoluted and specialized. The epithelium is folded into villi and the epithelial cells have an apical aspect covered by a dense matting of microvilli forming a brush border. This increases the small intestinal surface area for absorption by about 600 fold resulting in a higher capacity for nutrient absorption [7].

The aim of this study was to compare the gastrointestinal tracts of a recently bred genetic line of dual-purpose chicken, the Lohmann Dual, to a highly selected conventional broiler line, the Ross 308, to investigate specific morphological characteristics involved in intestinal digestion, resorption, secretion and barrier function. A better understanding of the gut development and anatomy in the two types of chickens provides new knowledge to improve feed efficiency and growth in the LD birds. Here, the allometric growth of the gastrointestinal segments were determined. The epithelium height, crypt depth, the height of intestinal villi, mucosal enlargement of the villus, and the thickness of the tunica muscularis of the intestine were measured morphometrically. The ultrastructure of the enterocytes and their interepithelial cell junctions were examined using transmission electron microscopy.

## Material and methods

### Birds and management

Two groups of 80 one day old male chicks, one a highly selected commercial broiler line (Ross 308) obtained from BWE-Brüterei-Weser-Ems GmbH & Co. KG, Visbek, Germany and the second a novel dual-purpose line (Lohmann dual, LD) were supplied by Lohmann Tierzucht, Cuxhaven, Germany. The rearing of the chickens was carried out in accordance with German animal welfare law. The study was approved by the Animal Welfare Committee: Landesamt für Gesundheit und Soziales, Berlin, Germany, ID: 0236/15.

The two groups were received at the same day in the Institute of Poultry Diseases of the Department of Veterinary Medicine, Freie Universität Berlin and housed under the same husbandry conditions. To reach the optimal body weight of 2000 g for slaughter the Ross chicks were kept for 5 weeks and the LD chicks for 9 weeks. Each group was kept in a separate 4.2 m^2^ pen in the same room having the same floor litter material. Both pens had a width of 1.63 m and a length of 2.55 m with 2.55 m high walls. The pens were separated by wire mesh. The photoperiod program used was 24 hours (h) light for the first three days, followed by 20 h of light until day 7 and 16 h of light thereafter. At the beginning of the study, temperature was kept at 30°C for three days, and was gradually reduced by 3°C per week until reaching 24°C. The desired temperature was regulated by a thermostat and the ventilation was controlled automatically, whereby the desired stable temperature was controlled by a thermostat. A mash diet was fed ad libitum to the two groups throughout the study and the birds were allowed free access to water. Birds were fed a starter diet from 1 to 14 days of age, then a grower diet from day 15 to the end of the study. The composition of the diet is shown in Table 1. On day one at the hatchery all birds received vaccinations against Newcastle disease and infectious bronchitis. Lohman dual birds also received vaccination against Marek’s disease. The birds were checked daily by a veterinarian. Samples from each group were collected at the following ages: Ross = 1, 7, 14, 19, 21, 25, 28, 32 and 35 day(s), LD = 1, 7, 14, 21, 28, 32, 35, 42, 49, 56 and 63 day(s). On sampling days, six birds were selected at random from the genetic line being examined, the live body weights were recorded, then the birds were killed by decapitation.

**Table 1:** Composition of diet feed.

Ingredients		Starter	Grower
Maize meal	%	23.95	29.13
Soybean meal	%	36.04	30.60
Wheat meal	%	28.00	28.00
Limestone	%	1.54	1.60
Monocalcium phosphate	%	1.2	0.85
^1^ Premix	%	1.2	1.2
DL-Methionine	%	0.27	0.25
L-Lysine HCl	%	0.15	0.17
Soybean oil	%	7.65	8.2
**Analyzed nutrient content of the (g or mg/kg in feed)**			
ME_N_ ^2^	MJ/kg	12.60	13.00
Dry matter	g/kg Us	896.43	894.65
Crude ash	g/kg Us	55.57	50.16
Crude protein	g/kg Us	231.5	214.4
Crude fat	g/kg Us	98.83	97.08
Crude fibre	g/kg Us	55.94	53.12
Starch	g/kg Us	284.09	334.67
Potassium	g/kg Us	8.20	7.44
phosphorus	g/kg Us	2.33	2.16
Calium	g/kg Us	8.20	7.24
Sodium	g/kg Us	1.57	1.43
Magnesium	g/kg Us	2.36	2.29
Iron	mg/kg Us	348.05	320.69
Manganese	mg/kg Us	113.44	103.2
Copper	mg/kg Us	20.94	19.39
Zinc	mg/kg Us	98.63	92.55

^1^Contents per kg premix: 600000 I.U. Vit. A (acetate); 120000 I.U. Vit. D3; 6000 mg Vit. E (α-tocopherol acetate); 200 mg Vit. K (MSB); 250 mg Vit. B1 (mononitrate); 420 mg Vit. B2 (cryst. Riboflavin); 300 mg Vit. B6 (pyridoxin-HCL); 1500 μg Vit. B12; 3000 mg niacin (niacin amide); 12500 μg biotin (commercial, feed grade); 100 mg folic acid (cryst., commercial, feed grade); 1000 mg pantothenic acid (Ca d-pantothenate); 60000 mg choline (chloride); 5000 mg iron (iron carbonate); 5000 mg zinc (zinc sulfate); 6000 mg manganese (manganous oxide); 1000 mg copper (copper oxide); 45 mg iodine (calcium-iodate); 20 mg selenium (sodium-selenite); 140 g sodium (NaCl); 55 g magnesium (magnesium sulphate); carrier:calcium carbonate (calcium min 38%).

^2^Estimated according to equation of WSPA 1984.

### Macroscopic examination

Directly after death, the abdominal cavity was opened by a ventral ceolomic incision, and the glandular stomach, the gizzard and the entire intestine of each bird were excised. The length of each intestinal segment was measured, i.e., duodenum (from the gizzard junction to the end of the pancreatic loop), jejunum (from the aboral pancreatic loop to Meckel’s diverticulum), ileum (from Meckel’s diverticulum to the ileocecal junction), and colorectum (from the ileocecal junction to the orad cloaca) as defined by De Verdal et al. (2010) [4]. Care was taken to avoid stretching the intestine. All adherent anatomical structures (i.e. mesentery, associated blood vessels, fat and pancreas) were removed from each organ at the time of collection. Organs were kept in Ringer’s solution for transport and processing.

In the laboratory, the glandular stomach, gizzard and the entire intestine including the ceca for each bird were opened longitudinally along the antimesenteric side, flushed with tap water to remove the contents, and blotted dry using filter paper. The weights of the glandular stomach, gizzard, and intestine segments were determined using an electronic laboratory balance (AND HF-200G, Tokyo, Japan) with a measurement accuracy of 0.01g. The normalized mass of the glandular stomach, gizzard and intestinal segments was calculated as [mass (g)/total body weight (g)] × 100. The weekly weight gain of the glandular stomach, gizzard and the intestinal segments compared with their final mass was calculated using the following relationship: [(the mean mass in the present week − the mean mass in the previous week)/ the final mean mass on the last day of the study) × 100]. The relative length of the intestine was calculated as [intestine length (cm)/total body weight (g)] × 100. The intestine mass per unit of length (g/cm) was defined as the “intestinal density” [8].

### Sample collection and histological examination

Samples (size 1x1x1 cm) of jejunum (1 cm proximal to Meckel’s diverticulum) and ileum (1 cm proximal to the ileocecal junction) were excised and fixed in neutral buffered formalin (4%, pH 7, 24 h, 20–24 °C). Then the tissues were dehydrated in an ascending graded series of ethanol and embedded in paraffin wax. Serial sections were cut at 5 μm with a microtome (Jung, Histoslide, 2000 Sliding, Wetzlar, Germany). Four cross-sections of jejunum and ileum per bird were stained with Meyer’s Hematoxylin and Eosin (H&E) according to standard histological protocol [9].

### Specimen preparation for electron microscopic examination

Samples of the jejunum (2 cm proximal to Meckel’s diverticulum) collected from two Ross 308 birds and two LD birds on days 1, 7, 21, and 35, and from the LD group on day 63, were fixed in Karnovsky solution (7.5% glutaraldehyde and 3% paraformaldehyde in phosphate buffered saline), washed in 0,1 M cacodylate buffer (cacodylic acid sodium salt trihydrate, Roth; Karlsruhe, Germany), incubated in 1% osmium tetroxide (Chempur; Karlsruhe, Germany) for 120 min., dehydrated in an ascending series of ethanol and washed in the intermedium propylene oxide (1, 2 Epoxypropan; VWR, Germany). Then the specimens were embedded in a mixture of agar 100 (epoxy resin), DDSA (softener), MNA (hardener) and DMP 30 (catalyst) (all: Agar Scientific; Stansted, GBR). Polymerization was done at 45°C and 55°C, each for 24 hours. Semi- and ultrathin sections were cut on an ultramicrotome Reichert Ultracut S (Leica; Wetzlar, Germany). Semi-thin sections (0.5 μm) were stained according to a modified Richardson protocol [9] for 45 seconds on an electric hotplate adjusted to 80°C and checked under a light microscope (Olympus CX 21, Olympus; Stuttgart, Germany) to determine the area of interest. Ultrathin (80 nm) sections were mounted on nickel-grids (Agar Scientific; Stansted, GBR) and examined with a transmission electron microscope (Zeiss EM 900; Oberkochen, Germany) (TEM).

### Morphometric analysis

For each bird, the mucosal and muscular layers of the jejunum and ileum were examined using a light microscope (Axioskop, Carl Zeiss, Jena, Germany) and an analyzing system, NIS-Elements AR (Nikon Instruments Inc., U.S.A.).

**Fig 1:**
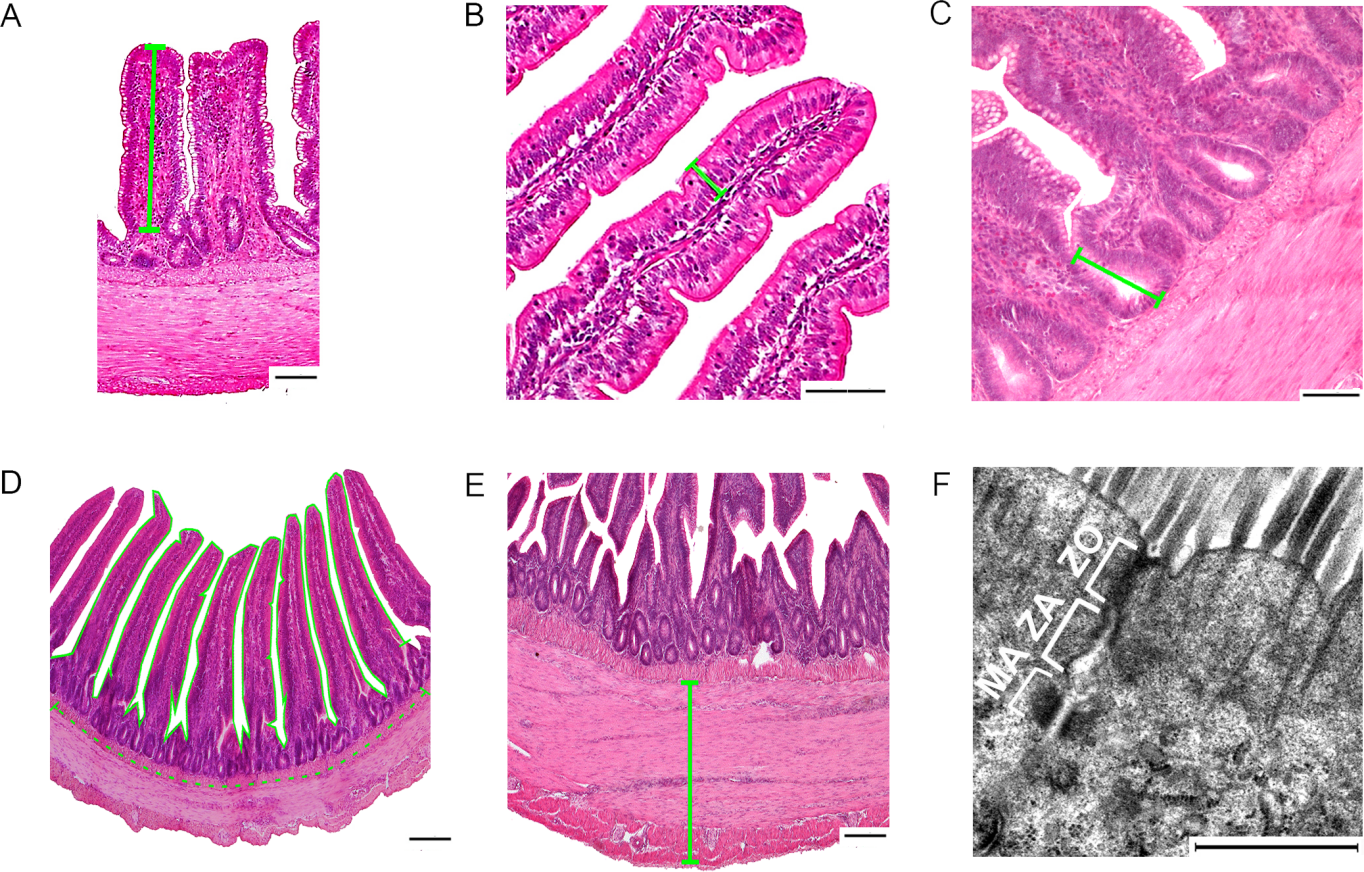
Morphometric measurements. (A) ileal villus height, (B) jejunal epithelium height, (C) crypt depth, (D) enlargement factor of the mucosal surface of the jejunum (The continuous line is the mucosal surface of the villi length and the interrupted line is the lamina muscularis mucosae length, (E) thickness of the tunica muscularis of the jejunum. Bar: 100 μm for A, B, C, D and E. (F) transmission electron micrograph of the enterocyte junctional complex. Here: ZO, zonula occludens; ZA: zonula adhaerens; MA, macula adhaerens (desmosome). Bar: 2000 nm.

The following parameters in H&E stained sections were measured on one cross-section per bird and intestinal segment:

- Villus height: Ten villi were measured from their base at the level of the crypt’s entrance through to their distal tips. Only full finger-shaped and well-oriented villi were used (Fig 1A).
- Epithelium height: Ten jejunal epithelial cells of different villi were measured from the basement membrane to the tip of their microvilli (Fig 1B).
- Crypt depth: Ten crypts were measured from the crypt’s base to the closest villus base. The ratio of villus height to crypt depth was calculated by dividing villus height by crypt depth (Fig 1C).
- Mucosal enlargement factor of the villus: Here the continuous length of the mucosal surface of ten adjacent villi was measured. The length of the corresponding underlying lamina muscularis mucosae was measured [10] (Fig 1D). Mucosal enlargement factor = total mucosal surface length divided by lamina muscularis mucosae length.
- Thickness of the tunica muscularis: This parameter was defined as the distance between the lamina muscularis mucosae internally and the tunica serosa externally. Ten measurements were performed per intestinal segment (Fig 1E).

The following parameters were measured in each jejunal sample using transmission electron micrographs.

- Microvillus length: Forty individual microvilli were measured from the tip of the microvillus to its attachment to the enterocyte membrane.
- Junctional complex and tight junction length: Ten enterocyte junctional complexes were measured from the apex of the tight junction to the innermost part of the desmosome. The length of each tight junction was measured from its apex to the start of the zonula adherens on the electron micrographs for each bird (Fig 1F).

### Statistical analysis

Data were analyzed using the statistical package program IBM SPSS Statistics 23 (IBM Corporation, New York, USA). The graphs were made by using the statistical package program JMP® Pro 13 (SAS Institute Inc., Cary, USA). Continuous variables are presented as standard error of the mean ± (SEM). Comparisons of the two lines of the same age groups were performed using the Mann–Whitney U test. The relationship between the normalized mass for each part of the stomach, intestinal segments and the relative length of the intestinal segments and age was assessed using one-way analysis of variance (ANOVA) with the post hoc Dunnett’s test. To explore the effect of chicken line and body weight on the glandular stomach and gizzard and intestinal segments, all data collected were regressed against the genetic line and body weight using the log-log regression model. Due to the non-linear relationship between the glandular stomach mass, gizzard mass, and intestinal segment masses to body weight, the data were log_10_-transformed prior to analysis. All statistical analyses were two-sided with significance defined as a p value of < 0.05.

To examine whether the relative changes of the glandular and gizzard and intestinal segments masses were proportionate or disproportionate to overall body weight relative change, the allometric relationships between the glandular and gizzard and intestinal segments and body weight were determined based on the following relationship [11]:

ln y = k ln x + ln b, where ln natural logarithm, y is the mass of the glandular stomach, gizzard or intestinal segments, x is the weight of the bird, b is a constant reflecting the relationship between the mass of the stomach parts and intestinal segments and the total body weight of the bird. The symbol k is the slope of the regression line relating y and x and represents the rate of change of the stomach parts and intestinal segments with changes in the total body weight. If the value of k is equal to 1 (isometry), the rate of change of the glandular and gizzard and intestinal segments with changes in the total body weight is proportionate during the growth. If k departs significantly from a value of 1, then the relationship is allometric (k >1 = positive allometry; k< 1 = negative allometry).

## Results

### Total body weight

From d 1 to d 35 post hatching, the body weight of the Ross birds increased at a rate of 57.67 g/d, whereas that of the LD birds increased by 22.03 g/d. The LD birds’ weight increased by a rate of 31.76 g/d from d 1 to d 63 post hatching. When compared with the final body weight of each genetic line the greatest body weight gain of 39.35% was between day 28 and 35 for Ross birds and of 19.48% between day 42 and 49 for LD birds.

**Fig 2:**
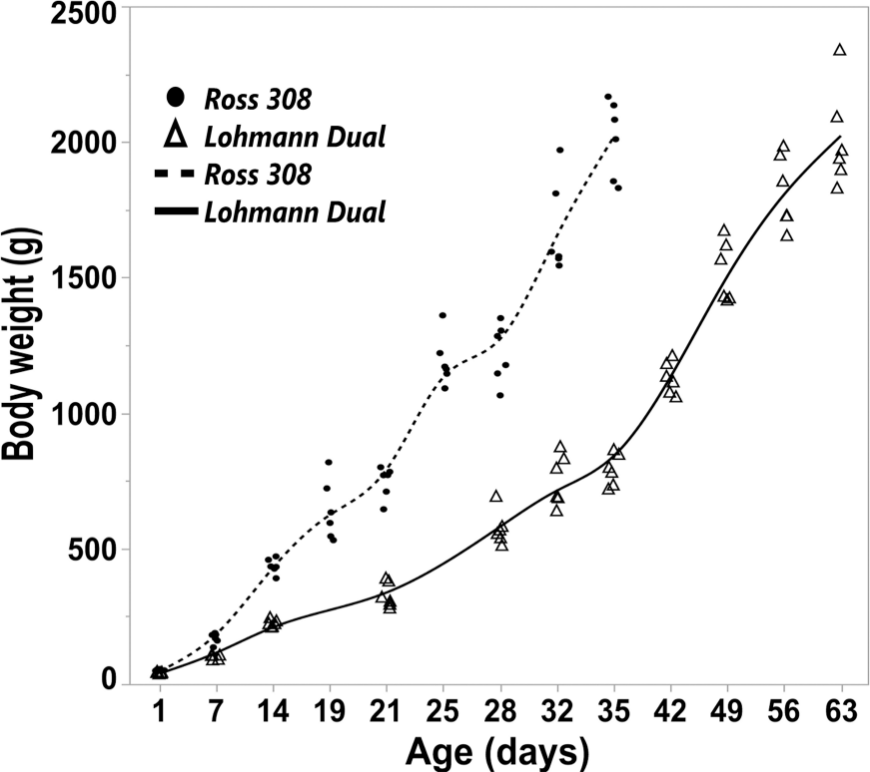
Trendlines of the changes in body weight versus day post hatching for Ross and LD chicken genetic lines. Symbols represent each individual value for each chicken line.

The Ross birds were about 2.6 times heavier than the LD birds by d 35 (Ross = 2013.16 g, LD = 791.66 g) (Fig 2).

### The glandular stomach

**Fig 3:**
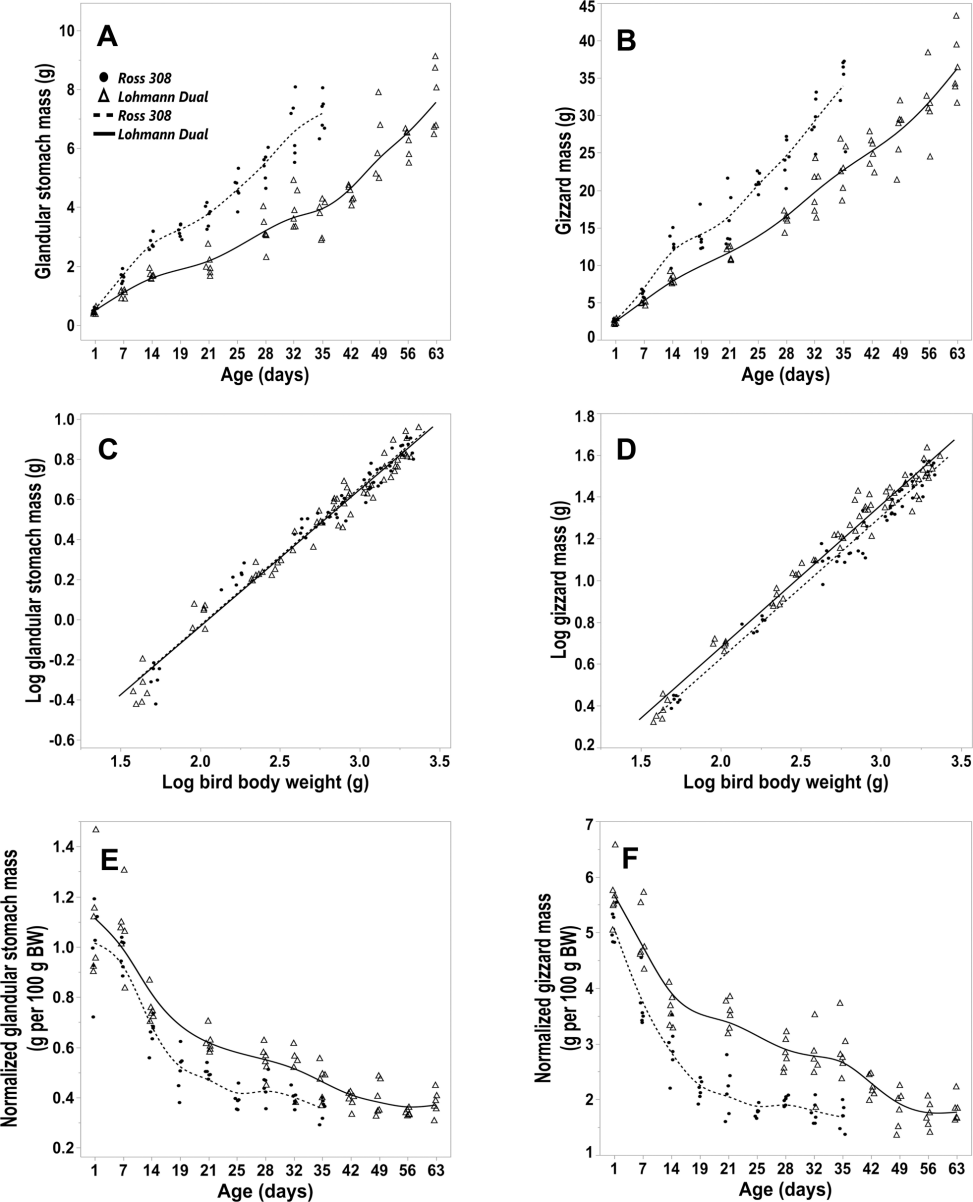
Morphometric parameters of the glandular stomach and gizzard for LD and Ross 308 chicken lines. (A, B) glandular stomach and gizzard mass versus day post hatching for Ross and LD lines. (C, D) allometric plot: logarithm (Log_10_) of glandular and gizzard mass versus (Log_10_) of total body weight for both genetic lines. (E, F) normalized glandular and gizzard mass versus day post hatching for both genetic lines. Symbols represent each individual value for each chicken line.

The mass of the glandular stomach of Ross birds increased from d 1 to d 35 post-hatching at a rate of 0.19 g/d, whereas that of LD birds increased at a rate of 0.09 g/d. The glandular stomach mass of LD birds from d 1 to d 63 increased at a rate of 0.11 g/d (Fig 3A). The growth of the glandular stomach was nearly isometric until d 7 in both lines. From d 14 onward, the glandular stomach exhibited negative allometric growth relative to body weight (Table 2) (Fig 3C). Regression analysis showed that the genetic chicken line had no influence on the mass of the glandular stomach.

**Table 2:** Allometric relationship between body weight and the mass of the glandular stomach, gizzard and entire intestine of both chicken lines for different post hatching growth periods.

	Period (days)	Line (n)	Unstandardized coefficients						
			Constant			Slope			R^2^
			Mean	LCI	UCI	Mean	LCI	UCI	
**Glandular stomach**	**1 to 7**	**Ross (12)**	-1.97	-2.26	-1.68	0.98	0.84	1.12	0.96
		**LD (12)**	-1.92	-2.36	-1.48	0.97	0.73	1.21	0.89
	**1 to 35**	**Ross (54)**	-1.4	-1.5	-1.3	0.69	0.65	0.72	0.97
		**LD (36)**	-1.44	-1.55	-1.33	0.7	0.66	0.75	0.96
	**1 to 63**	**LD (66)**	-1.4	-1.48	-1.32	0.68	0.65	0.71	0.97
**Gizzard**	**1 to 7**	**Ross (12)**	-0.79	-0.94	-0.63	0.71	0.63	0.79	0.97
		**LD (12)**	-0.96	-1.21	-0.71	0.83	0.69	0.96	0.94
	**1 to 35**	**Ross (54)**	-0.73	-0.82	-0.65	0.68	0.65	0.71	0.98
		**LD (36)**	-0.8	0.89	0.71	-0.73	0.7	0.77	0.98
	**1 to 63**	**LD (66)**	-0.68	-0.76	-0.61	0.68	0.65	0.71	0.97
**Entire intestine**	**1 to 7**	**Ross (12)**	-1.52	-1.82	-1.22	1.13	0.98	1.29	0.97
		**LD (12)**	-1.99	-2.29	-1.69	1.43	1.27	1.59	0.97
	**1 to 35**	**Ross (54)**	-1.1	-1.18	-1.03	0.92	0.89	0.95	0.99
		**LD (36)**	-1.12	-1.23	-1.01	0.94	0.89	0.98	0.98
	**1 to 63**	**LD (66)**	-0.94	-1.03	-0.85	0.85	0.82	0.89	0.98

LCI: lower confidence interval; LD: Lohmann dual; n: animal number; R^2^: coefficient of determination; Ross: Ross 308; slope: this value represents the allometric relationship between the dependent variables and body weight; UCI: upper confidence interval.

From d 1 to d 35 post-hatching, the normalized mass of the glandular stomach was numerically greater in the LD line than in the Ross line in all age groups. The difference was statistically significant from d 14 onward. A spike in the normalized mass of the glandular stomach was noted on d 1 for LD and Ross chicks at values of 1.09 and 1.00 g per 100 g BW, respectively (Fig 3E). Then, there was a decrease in the normalized glandular stomach mass with age until d 25 for Ross and d 35 for LD chicks. Subsequently the decrease was insignificant until the end of the study. Furthermore, the post hoc Dunnett’s test indicated no differences between each age group from d 25 for Ross chicks and from d 32 for LD chicks compared with the last day of the study.

### The gizzard

The gizzard grew gradually with age in both lines, with the mass of the gizzard increasing by 12.65 times in Ross birds and 15.15 times in LD birds by the end of the study (Fig 3B).

The gizzard of Ross chicks gained 0.91 g/d from d 1 to d 35 post-hatching (R^2^ = 0.91), whereas the LD chick’s gizzard gained 0.60 g/d over the same period. From d 1 to d 63, the gizzard mass of LD chicks increased at a rate of 0.55 g/d (R^2^ = 0.94). Overall, the gizzard had negative allometric growth relative to body weight (Table 2) (Fig 3D).

Regression analysis showed that both the BW and the genetic line of the chickens had an influence on the mass of the gizzard, p ≤ 0.001, adjusted R^2^ = 0.97. In birds of the same body weight, the gizzard mass of LD birds was heavier by 5.5% than that of Ross birds.

From d 1 to d 35 post hatching, the normalized mass of the gizzard was greater in LD birds than in Ross birds and the difference was statistically significant from the first day onwards (Fig 3F).

The normalized gizzard mass was greatest on the first day post-hatching, followed by a marked decrease until d 21 in both genetic lines. After this there was a gradual decrease until the end of the study. The post hoc Dunnett’s test showed that there were no significant differences between each age group from day 21 for Ross birds and from d 42 for LD birds compared with the last day of the study.

### The intestine

**Fig 4:**
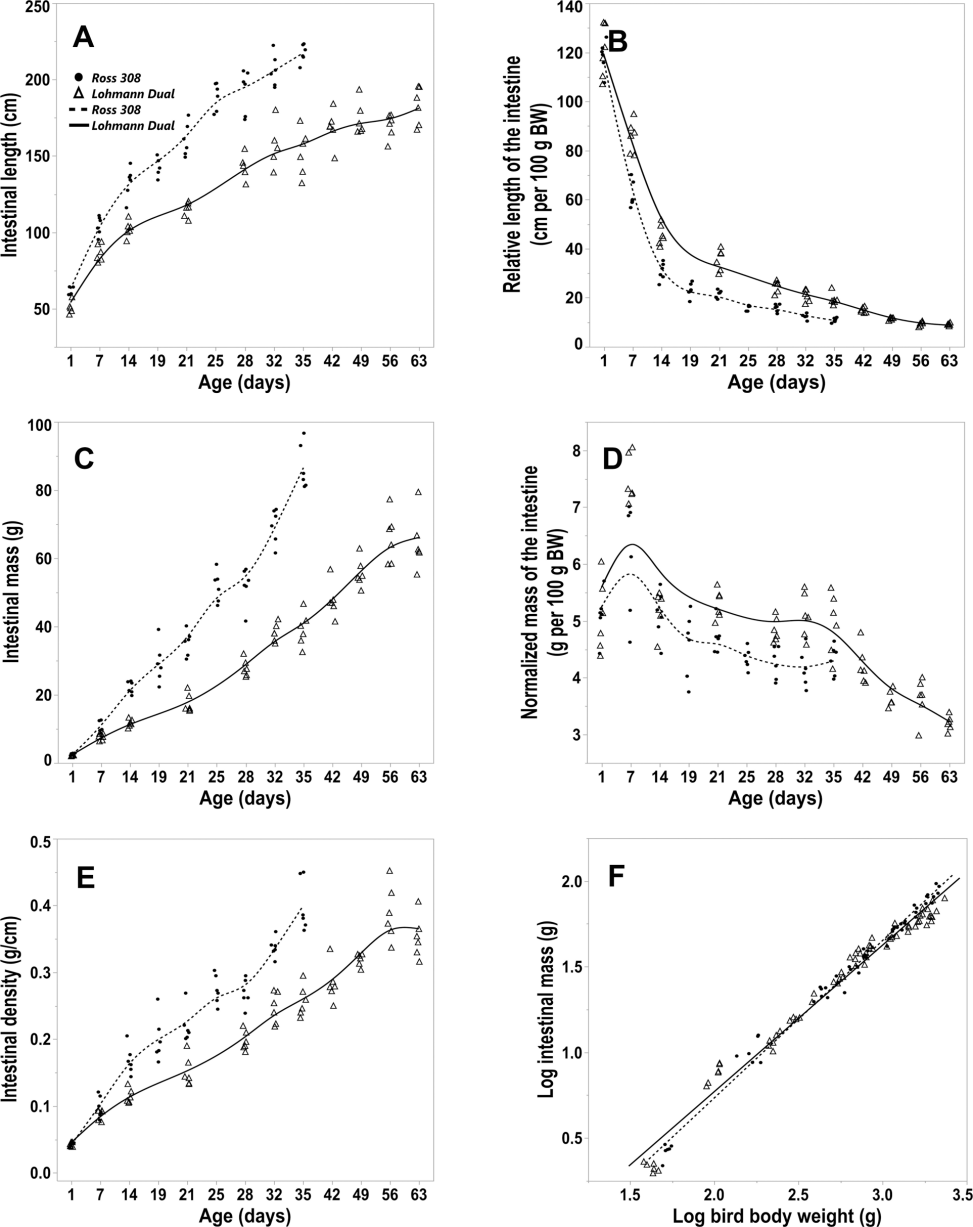
Morphometric parameters of the intestine for LD and Ross 308 chicken lines. (A) length of the entire intestine, (B) relative length of the entire intestine, (C) mass of the entire intestine, (D) normalized intestine mass, (E) intestinal density, all versus days post hatching. (F) allometric plot: logarithm (Log_10_) of the entire intestine mass versus Log_10_ of total body weight. Symbols represent each individual value for each chicken line.

The length and mass of the entire intestine increased from d 1 to d 35 post-hatching, in Ross chicks at rates of 4.57 cm/d and 2.47 g/d, and in LD birds at rates of 2.98 cm/d and 1.08 g/d respectively. The entire intestine of Ross chicks grew faster than it did in LD birds over this period i.e.,1.53 times in length and 2.28 times in mass (Fig 4A and 4C). Between d 1 and d 63 the daily rate of increase in the intestinal length and mass in LD chicks were 2.13 cm and 1.01 g respectively. The greatest increase in intestinal length was in the first week post-hatching i.e. by 19.5% of the final mean length in both lines. The mean of the entire intestine mass on d 1 post-hatching was 3.08% (Ross birds) and 3.31% (LD birds) of the mean of the final intestinal mass. The greatest weight increase of the intestine was between day 21 and 35 in both lines. The allometric relationship between BW and intestinal mass was positive until day 7 in both genetic lines and became negative from day 14 onward (Table 2) (Fig 4F).

According to regression analysis, both the BW and the genetic line of the chickens had an influence on the entire intestine length, p ≤ 0.001, adjusted R^2^ = 0.95. The entire intestine length of Ross birds was longer on average by 4% than that of LD birds of the same BW. However, only the BW had a significant influence on the entire intestine mass, p ≤ 0.001, adjusted R^2^ = 0.98. The normalized mass of the entire intestine was significantly greater from day 21 onwards in the LD birds than in Ross birds. The normalized intestinal mass showed that the two genetic lines were similar, peaking at day 7 then decreasing to plateau between days 21–35 (Fig 4D), with levels in LD subsequently dropping noticeably between days 35 and 63. The post hoc Dunnett’s test showed that there were no significant differences in the normalized entire intestine mass between the age groups from day 19 for Ross birds and from day 49 for LD birds compared with the last day of the study of each respective genetic line. From day 7 onwards the relative length of the entire intestine was significantly greater in LD birds than in Ross birds at the same age. The relative length of the entire intestine dropped sharply post-hatching until d 14 in both lines, where the mean values were 0.26 times and 0.38 times smaller than the value on d 1 for Ross birds and LD birds respectively. The relative intestinal length stabilised from d 25 onwards for Ross birds and from d 42 for LD birds to the last day of the study in each genetic line (Fig 4B). The post hoc Dunnett’s test showed no differences in the relative entire intestine length between the age groups from d 25 for Ross birds and from d 42 for LD birds compared with values on the last day in each genetic line.

The intestinal density increased continually with age in both lines (Fig 4E). The intestinal density of Ross birds was significantly greater than that of LD birds from d 14 onward Regression analysis showed that both BW and the genetic line of the chickens had an influence on the intestinal density, p ≤ 0.001, adjusted R^2^ = 0.97. In birds of equivalent BW the intestinal density of LD birds was higher on average by 3% than that of Ross birds.

#### Intestinal segments

The analysis of the normalized mass and relative length of the individual intestinal segments mirrored those determined for the entire intestine. The density (g/cm) of the individual intestinal segments in both groups was highest in the duodenum and gradually decreased aborally to be least in the colorectum (Table 3). The length and mass of each of the intestinal segments is reported as a percentage of the entire intestine in Table 3. Regression analysis for each intestinal segment showed that the genetic line of the chickens had a significant influence only on the jejunoileum and colorectum. The jejunoileum of Ross birds was 6.2% longer (p ≤ 0.001) and 2.8% heavier (p = 0.015) than LD birds of equal BW adjusted R^2^ = 0.93 and R^2^ = 0.98, respectively. In contrast, the colorectum was 3.1% heavier in LD birds than in Ross birds of the same body weight, p = 0.015, adjusted R^2^ = 0.97.

**Table 3:** Intestinal density and length and mass of individual intestine segments as a percentage of the entire intestine of both chicken lines over the study period.

Intestinal segments	Line (n)	Intestinal density (g/cm)		Length (%)		Mass (%)	
		Mean	SEM	Mean	SEM	Mean	SEM
**Duodenum**	**Ross (54)**	0.29	0.02	15.04	0.3	19.2	0.32
	**LD (66)**	0.29	0.02	16.76	0.19	22.66	0.46
**Jejunum**	**Ross (54)**	0.24	0.02	33.17	0.3	33.88	0.45
	**LD (66)**	0.25	0.02	32.63	0.19	35.64	0.36
**Ileum**	**Ross (54)**	0.22	0.01	31.12	0.35	30.23	0.43
	**LD (66)**	0.2	0.01	28.59	0.22	25.78	0.32
**Cecum**	**Ross (54)**	0.16	0.01	15.9	0.17	11.77	0.28
	**LD (66)**	0.14	0.01	16.52	0.17	10.53	0.21
**Colorectum**	**Ross (54)**	0.06	0.003	4.76	0.07	4.92	0.19
	**LD (66)**	0.07	0.004	5.49	0.07	5.38	0.12

Intestinal density: intestine mass per unit of length; LD: Lohmann dual; Ross: Ross 308; length (%) and mass (%): the length and mass of individual intestinal segment as a percentage of the entire intestine; n: animal number; SEM: standard error of the mean.

### Intestinal histology

**Fig 5:**
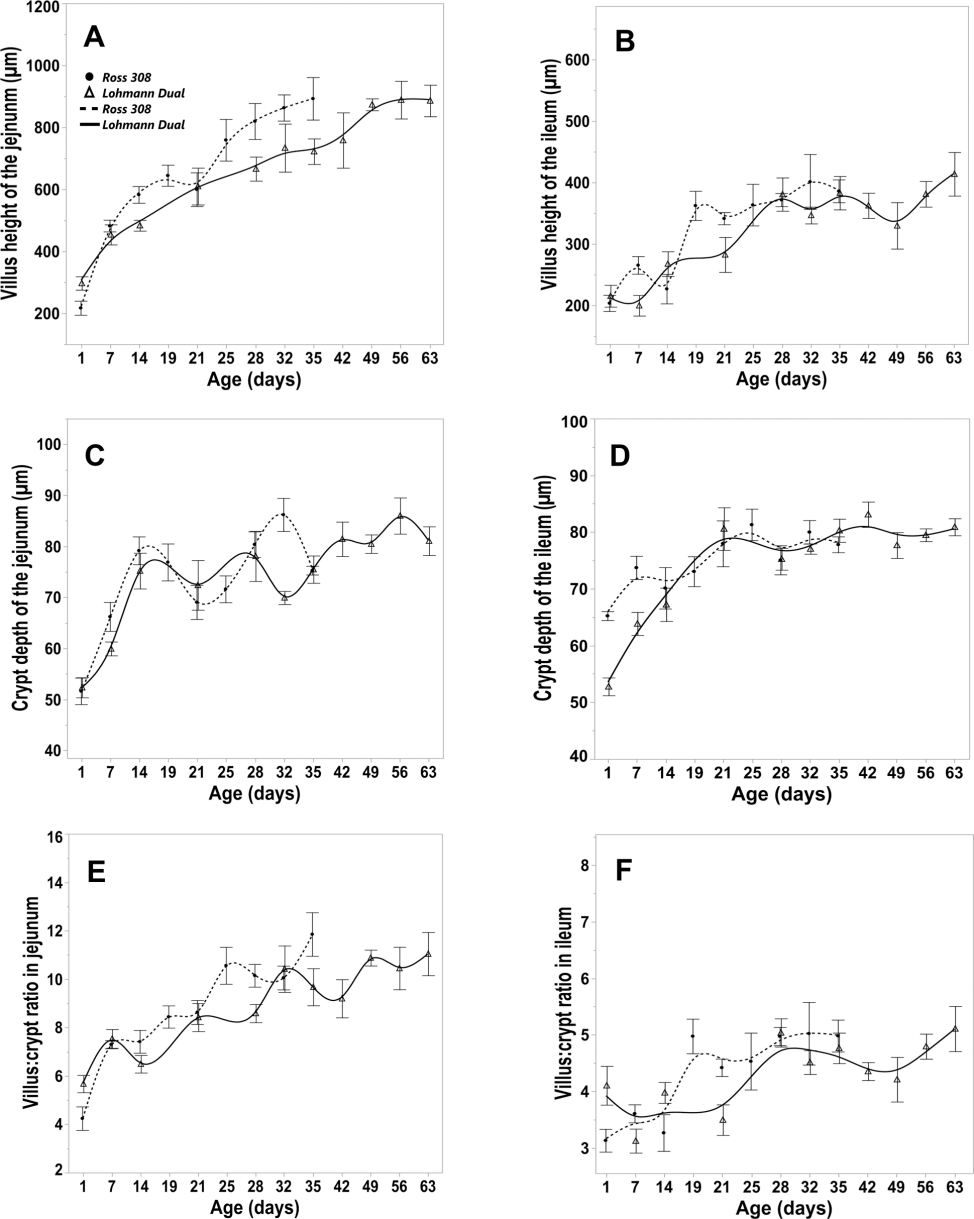
Morphometric parameters of the intestinal tunica mucosa for LD and Ross 308 chicken lines. Trendlines of changes in the villus height of the jejunum (A) and ileum (B), crypt depth of the jejunum (C), crypt depth of the ileum (D), villus crypt ratio in jejunum (E), villus crypt ratio in ileum (F). Bars refer to mean ± standard error of the mean of the sampled chicken at each time interval.

Jejunal villi had a finger-like appearance in birds of all ages in the study (Fig 1D). Ileal villi were shorter and had a more blunt appearance than those of the jejunum (Fig 1A). The height of the jejunal villus increased gradually with age in both genetic lines (Fig 5A). At d 35, the jejunal villus heights in Ross birds were 4 times higher than those at day 1. Whilst the jejunal villus heights in LD birds at day 63 were about 3 times higher than those of the one day old hatchlings. The greatest increase in jejunal villus height was from d 1 to d 7 in both genetic lines. On d 1, the jejunal villus heights were greater in LD birds than in Ross birds, however, by day 14 they were greater in Ross birds than in LD birds. Between d 21 and d 35 there was no difference between the two genetic lines (Fig 5A). The ileal villi were shorter than the jejunal villi in both genetic lines (Fig 5A and 5B). On the last day of the study, the ileal villus height was about 2 times greater than on the first day in both genetic lines. There were no significant differences in the ileal villus height between the age groups in both genetic lines (Fig 5B).

Regression analysis showed that the BW had a significant influence on the villus height of specific intestinal segments and that the genetic line of the chickens had an influence only on the jejunal villus height, p = 0.012, adjusted R^2^ = 0.76. The jejunal villus height of LD birds was, on average, 4% larger than in Ross birds of the same BW.

In the first week of life, there was a marked increase in the jejunal and ileal crypt depth in both genetic lines that continued until day 14 in the jejunum but not in the ileum (Fig 5C and 5D). From then until the end of the study changes in crypt depths were insignificant. The crypt depth of the LD birds was smaller than those of the Ross birds at day 1 and 7 in the ileum and at day 32 in the jejunum.

The villus to crypt ratio increased gradually with age in both genetic lines and was greater in the jejunum than in the ileum (Fig 5E and 5F). At d 1 the jejunal ratio was significantly greater in LD birds than in Ross birds, whereas by d 28 this ratio was greater (p < 0.05) in Ross chickens. There was no significant line difference in the ileal ratio of both genetic lines between the age matched groups.

**Fig 6:**
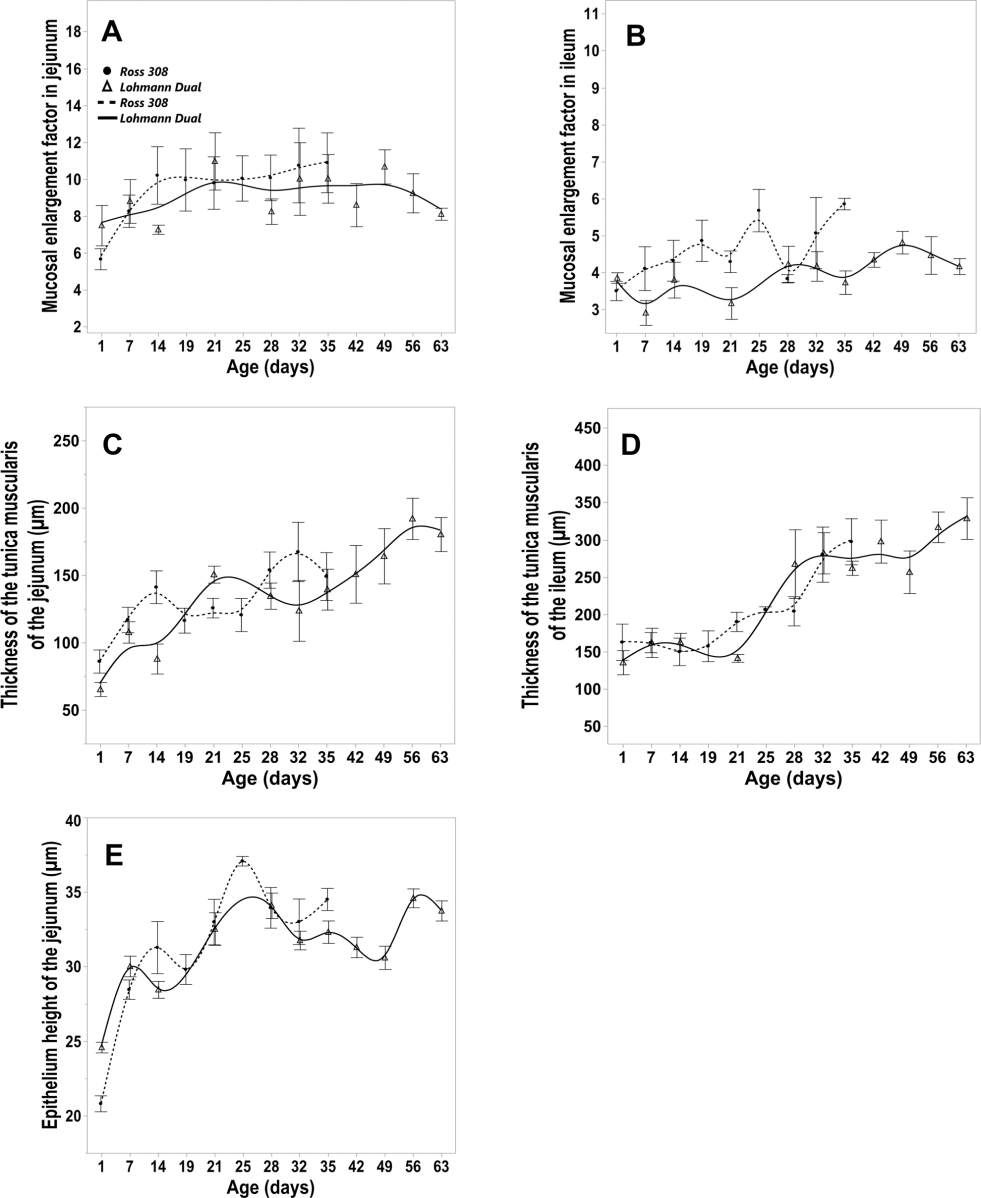
Morphometric parameters of the intestinal tunica mucosa and tunica muscularis for LD and Ross 308 chicken lines. Trendlines of changes in (A, B) mucosal enlargement factor in jejunum (A) and ileum (B), thickness of the tunica muscularis of the jejunum (C) and ileum (D), epithelium height of the jejunum (E). Bars refer to mean ± standard error of the mean of the sampled chicken at each time interval.

The jejunal epithelial height was significantly higher in LD birds (24.58 μm ± 0.87) than in Ross birds (20.81 μm ± 1.3) at day 1 but not thereafter (Fig 6E).

The mucosal enlargement factor of the jejunum was greater than that of the ileum in both genetic lines over the study period (Fig 6A and 6B). There was no difference in the mucosal enlargement factor of both genetic lines between matched age groups. Regression analysis showed that BW had a significant influence on the mucosal enlargement factor in jejunal and ileal segments and that the genetic line of the chickens had an influence only on the villus enlargement factor of the ileum, p = 0.006, adjusted R^2^ = 0.22. In birds of the same BW, the ileal enlargement factor of Ross birds was, on average, 5.6% greater than that of LD birds.

Independent of genetic line, the tela submucosa of the intestinal wall was weakly developed and appeared to be almost non existent (Fig 1A, 1C and 1E). The tunica muscularis consisted of a thick inner circular layer and a thin outer longitudinal layer. The thickness of the tunica muscularis was greater in the ileum than in the jejunum in all birds sampled throughout the study (Fig 6C and 6D). Regression analysis showed that the BW had a significant influence on the thickness of the tunica muscularis in both intestinal segments and that the genetic line of the chickens had an influence only on the thickness of the tunica muscularis of the ileum, p = 0.001, adjusted R^2^ = 0.45. The thickness of the ileal tunica muscularis in LD birds was on average 7.8% greater than in Ross birds of equal BW.

### Transmission electron microscopy

**Fig 7:**
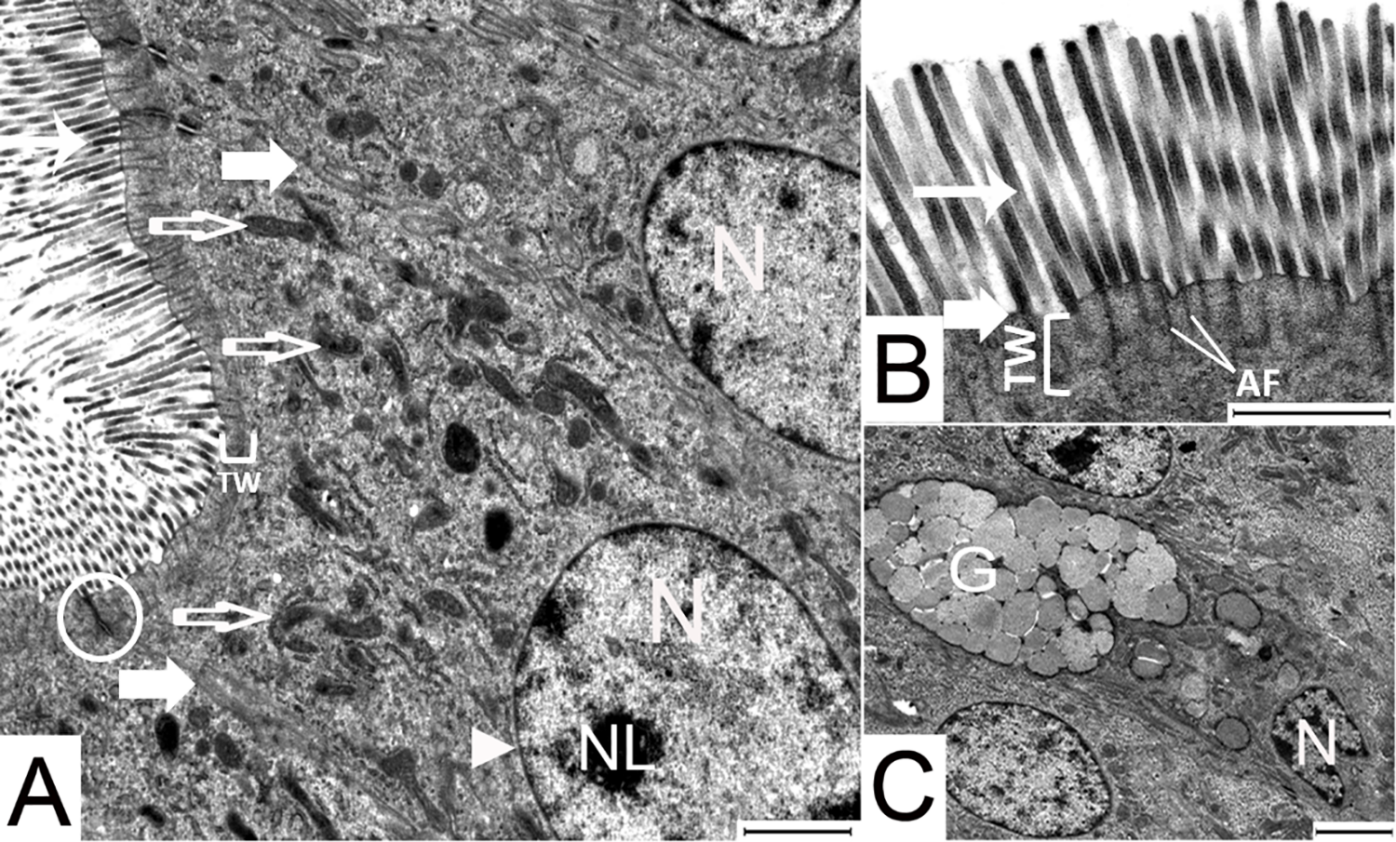
Transmission electron micrographs of jejunal enterocytes of 1 day old LD birds. (A) the organelles are arranged towards the apical end of the enterocyte, (B) microvilli, (C) goblet cell flanked on both sides by enterocytes. Arrow head: nuclear membrane; broad arrow, cellular membrane; circle, junctional complex; empty arrow, mitochondria; narrow arrow, microvilli; AF, actin filaments; G, secretory vesicles containing mucin; N, nucleus; NL, nucleolus; TW, terminal web. Bar: 2000 nm.

The transmission electron micrographs of jejunal samples of both genetic lines showed that the enterocytes interspersed with goblet cells were attached to the basement membrane. The enterocytes were rectangular in shape and were characterized by a dense layer of regularly arranged microvilli on their apical surface. The large euchromatic nuclei of the enterocytes were located basally and their organelles were arranged primarily in the apical compartment (Fig 7A).

The microvilli of the enterocytes extended from the apical part of the cell as finger-like shapes containing filamentous and glycocalyx formations in birds from both genetic lines at all ages (Fig 7B). From d 1 to d 35 the average microvillus length ranged from 2.04 μm to 2.58 μm. In the LD birds the microvillus length averaged 4.03 μm by day 63 (Table 4).

**Table 4:** Length of the microvillus, the tight junction and the junctional complex in the jejunum of both chicken lines versus day post hatching.

Age (days)	Line	Microvilli Length (μm)		Tight junction length (μm)		Junctional complex length (μm)		TJ:JC %	
		Mean	SEM	Mean	SEM	Mean	SEM	Mean	SEM
**1**	**Ross**	2.42	0.03	0.64	0.27	1.33	0.55	48.12	1.77
	**LD**	2.45	0.02	0.54	0.15	1.10	0.28	48.88	1.59
**7**	**Ross**	2.04	0.08	0.59	0.26	1.21	0.54	48.77	0.93
	**LD**	2.58	0.03	0.54	0.17	1.13	0.34	47.90	1.62
**21**	**Ross**	2.22	0.05	0.55	0.24	1.15	0.51	48.04	1.46
	**LD**	2.43	0.02	0.47	0.16	0.97	0.31	47.81	2.31
**35**	**Ross**	2.37	0.02	0.59	0.15	1.22	0.32	48.89	0.85
	**LD**	2.18	0.03	0.46	0.13	1.00	0.36	47.31	3.78
**63**	**LD**	4.03	0.04	0.53	0.15	1.09	0.29	48.04	1.83

JC: junctional complex; LD: Lohmann dual; Ross: Ross 308; SEM: standard error of the mean; TJ: tight junction; TJ:JC %: the tight junctions length as a percentage of the junctional complex length.

**Fig 8:**
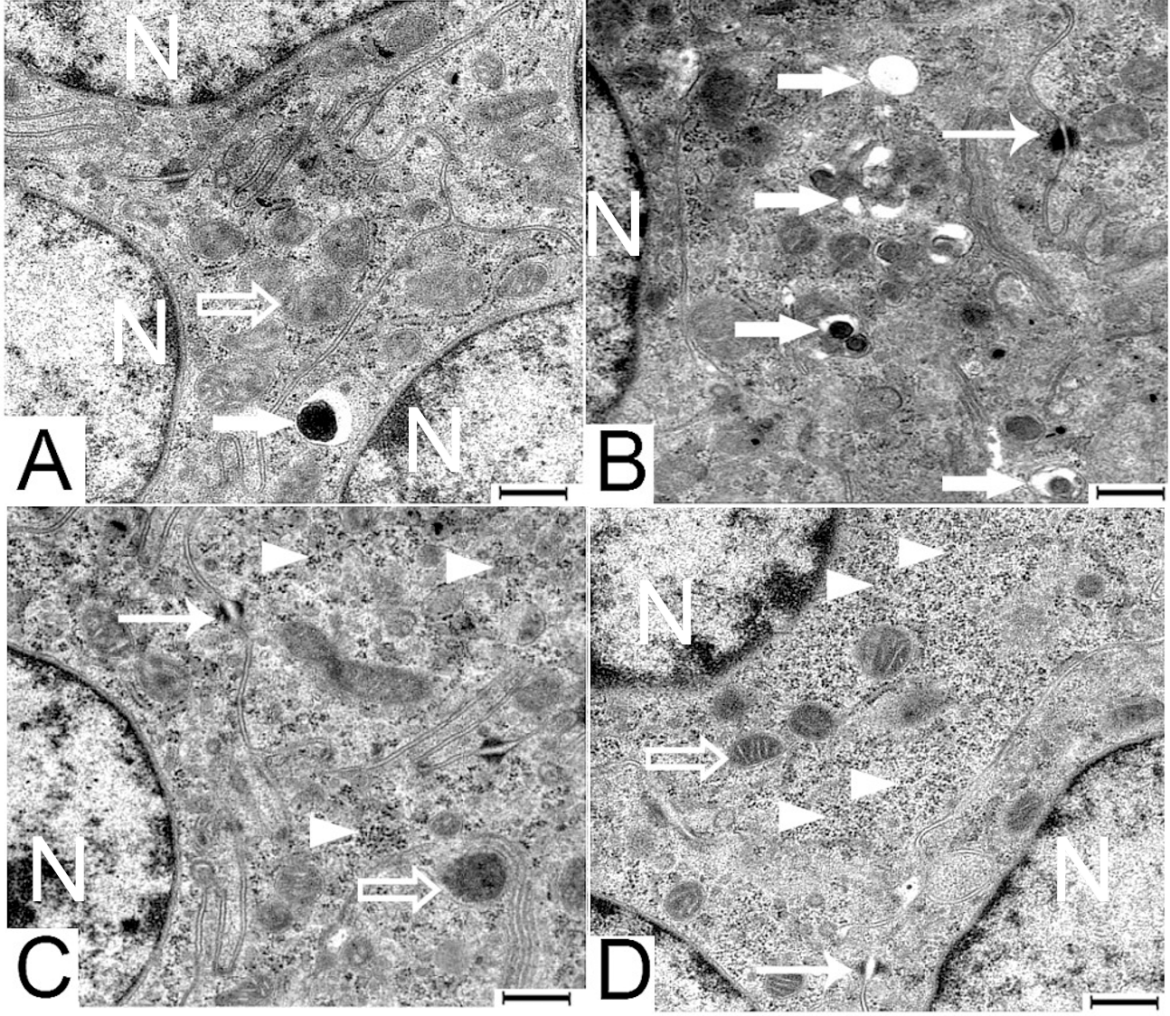
**Transmission electron micrographs of jejunal enterocytes of 1 day old LD [A and C] and Ross [B and D] chickens**. Arrow head, free ribosomes; broad arrow, lysosomes; empty arrow, mitochondria; narrow arrow, desmosomes; N, nucleus. Bar: 500 nm.

The junctional complexes in both genetic lines had a similar form and electron density. They occurred close to the lumen along the lateral surfaces of adjacent epithelial cells. Each consisted of three sequentially arranged structures; a tight junction (zonula occludens), an intermediate junction (zonula adherens) and a desmosome (macula adherens) (Fig 1F). The tight junction resides adjacent to where the plasma membrane reflects from the apical to the lateral surfaces of the enterocyte. It was characterized by fusion of the adjacent cell membranes over varying distances resulting in obliteration of the intercellular space. The intermediate junction linked the tight junction to the desmosome below. It was characterized by the presence of an intercellular space, of low density material. The desmosomes were characterized by a intercellular space having an electron dense core plus the presence of a surrounding electron-dense disc (Fig 1F; Fig 8B and 8C). Table 4 reports the mean length of the junctional complex and the tight junction. At all ages and in both genetic lines, the tight junction involved about 48% of the junctional complex length (Table 4).

The observations of the electron micrographs showed that, the enterocyte’s cytoplasm on day 1 contained many lysosomes and free ribosomes in both genetic lines. The enterocytes of the Ross birds appeared to have greater concentration of lysosomes (Fig 8A and 8B) and free ribosomes (Fig 8C and 8D) than those of LD birds. By day 7 the enterocyte’s lysosomes had disappeared and there were fewer free ribosomes. Mitochondria having different forms such as; rod-like, oval-shaped, and tadpole-shaped, were present throughout the time-line of the study in both genetic lines of the chicken. On day 1, the mitochondria were scattered throughout the cytoplasm. Subsequently by day 7 they were seen to be aggregated in the perinuclear region as well as in the apical part of the epithelial cells. Over time the number of mitochondria in the enterocytes increased in both chicken genetic lines.

The goblet cells are a simple columnar epithelial cell that were similar in size to their coexisting enterocytes. Their narrow bases were attached to the basement membrane and their cell bodies extended through to the lumen (Fig 7C). The cup shaped apical part of the goblet cells were filled with mucus granules of differing electron densities. Their ovoid nuclei were located basally in the narrow stem-like portion. Most other organelles lay between the nucleus and the mucus granules.

## Discussion

The breeding of dual production chicken lines with high performance females for laying eggs and males being raised for meat production is an alternative to the culling of one-day-old male chickens of laying lines. Lohmann Dual, a recent commercial dual-purpose breed developed by crossing meat and layer lines and using the sex-linked dwarf gene, has acceptable performances in both meat and egg production [3]. Over the 70 days of their life their feed conversion ratio is 1:2.5 which is better compared to that of Lohmann Brown cockerels 1:4 [12].

The highly selected broiler line (Ross 308) and the Lohmann Dual line (LD) reported in the present study had markedly different growth patterns in their body weight and in gastrointestinal segmental morphology over the time frame of the study. The mean BW of Ross 308 chicks was significantly higher than that of the LD chicks at all ages since Ross’s genetic makeup made it more prone to an increased BW compared to LD over a short time frame.

In 2015 Damme et al. examined the meat production of three commercial dual-purpose chicken lines; Lohmann Dual, Walesby Specials and Dominant Red Barred. After 84 days, the male chickens of the LD had a BW of 3165 g while the Walesby Specials and Dominant Red Barred had BWs of 2423 g and 1818 g, respectively [3]. However Habig et al. (2016) reported that the BW of the LD males reached only 1803 g after 77 days [13]. This contrasts dramatically with our finding that LD birds reached 2012 g by 63 days. These disparities may be a result of either different feed and husbandry conditions or the effect of individual differences in the growth rate in this chicken line as reported by Urban et al. (2018) [14].

A significant side effect of the intensive genetic selection of high performance meat production chicken lines has been a disruption in the balance between the relative slow growth of their organs and their extremely rapid increase in muscle mass [15–18]. Our results confirm this side effect where the normalized mass and relative length of the gastrointestinal of the slow growing LD birds were greater than those of the fast growing Ross chicken. The high normalized mass and great relative length of the gastrointestinal tract reported in this study of LD birds corresponds to those observed in slow growing chicken lines [4, 19, 20].

In this study the intestinal mass had positive allometric growth over the first week post-hatching but subsequently negative allometric growth for the remainder of the study period in both genetic lines. Similarly the greatest increase in jejunal villus height occurred over the same period. This reflects the chicken’s conversion from a reliance on yolk sac constituents to the components of their solid diet over the first week of life [21]. Subsequently throughout their life, the relative weight and length of their intestine decreased whilst overall villus heights increased. This most likely enables the chicken to absorb nutrients efficiently as their metabolic demands rise with increasing body mass over time [7]. When chickens get older, the small intestinal absorptive capacity depends more on increasing overall villus surface areas rather than increases in the intestinal length and mass [7].

From day 1 the relative intestinal length and from day 7 the normalized mass of the gastrointestinal segments decreased significantly in both lines until a specific time, after which the decrease became insignificant. This suggests that a balance between the growth rates of gastrointestinal segments to the body weight had been established at this time point. This balance occurred earlier in Ross chicks than in LD chicks where; normalized glandular stomach mass was 7 d later, normalized gizzard mass was 21 d later and intestinal relative length was 17 d later. Similar findings for gastrointestinal tract segments have been reported in four different genetic lines of ducks, where the maximal growth rate of the gastrointestinal segments was earlier in the fast-growing genetic lines than in the slow-growing genetic lines [22]. This suggests there is a temporal discrepancy between the development of the organs and the final body weight. The same phenomenon occurred between LD and Ross. Here the time-lines to reach the balance time of each gastrointestinal tract segment and reach the final body weight of 2000 g was different in each genetic line. To reach a BW of 2000 g LD birds needed 4 more weeks than Ross birds. In general, the different growth rates are due to a complex interaction of genetic, physiological and environmental factors including; diet composition, diet form, feeding strategy [23]. In this study, the genetic influence appears more relevant than others since both lines were maintained under similar conditions. Most likely these differences in body growth rates in our study may have anatomical reasons, since the normalized, stomach and intestinal segment masses as well as relative lengths of LD birds took 1–3 weeks longer in LD birds than in Ross birds to reach a stable level.

In this study regression analysis indicated that LD birds had a larger gizzard, shorter intestine and smaller mucosal enlargement factor than did Ross birds of the same BW. Recently a morphometric study on the gastrointestinal tract of two broiler chicken lines genetically selected for a high digestion (D+) or a low digestion (D−) efficiency was reported [4]. Although the D- birds consumed more feed and had a greater villous surface area than the D+ birds, the D+ birds had faster growth rates than the D− birds. However, the ability to digest starch, protein and lipids was shown to be lower in the D− compared with D+ line. The glandular stomach and gizzard were significantly lighter and the length and weight of the small intestine, villus width and surface area and the tunica muscularis thickness of the D- birds were significantly greater than in D+ birds. The authors suggested that the intestinal adaptations of the D− birds may be an attempt to compensate for the low functionality of their gastric area [4].

Taylor and Jones (2004) fed Ross 308 chickens for 42 days on a pelleted diet, of wheat or barley that was either whole grain or ground. They found that the whole grain diet reduced the relative mass of the glandular stomach but increased the relative mass of the gizzard. The birds fed whole grain diet had a highly viscous alkaline ileal digesta together with a decreased relative mass of each intestinal segment [24]. The authors suggested that an active gizzard increased starch availability in the intestine and that this is associated with enhanced peristaltic movement and influenced the mass of each intestinal segment [24].

The villus height in chickens decreases gradually from orad to aborad along the small intestine [25]. This is due to the greater absorptive capacity of the duodenum followed by that of the jejunum and then the ileal villi [25]. The intestinal histological modifications are mostly related to the availability and nature of the nutrients within the intestine, for example, the small villi can undergo compensatory enlargement due to the intestine having elevated nutrient levels over a period of two to five days [26, 27].

Our study supports the idea that the more muscular gizzard in the LD birds increases the degree of food processing and enhances the availability of nutrients in the proximal small intestine where gut plasticity is more pronounced [24, 28]. This is mirrored histologically by more elongated jejunal villi that in turn lead to the ileum receiving digesta having lower nutrient levels resulting in a decreased ileal absorptive surface area in LD birds compared to Ross birds of the same BW.

Because the tunica muscularis determines the rate and power of intestinal motility it governs the progression of a bolus and that in turn affects the absorption processes by increasing or decreasing the contact between the mucosa and the intestinal contents [4]. A thick tunica muscularis and a shorter intestine in LD birds could lead to a more rapid intestinal passage time and a lower uptake of available nutrients.

Whilst in our study the husbandry and nutrition were the same for both chicken lines there are many factors that can influence the growth rate of the gastrointestinal tract and its morphological characteristics. The differences between the two genetic lines in intestinal length and gizzard weight are probably due to genetic differences and are important criteria for ongoing selection for improved performance of the LD male birds.

In the current study epithelial cells of day old Ross chicks had greater concentration of lysosomes than did LD chicks. Yamauchi et al (1992) presumed that these lysosomes were residual bodies of lipid digestion derived from the yolk sac [29]. Noble and Cocchi 1990 reported that during the incubation period of the chick, 80% of the yolk lipid content is mobilized and absorbed by embryonic tissues. The metabolism of the yolk lipids continues after hatching and is sufficient for the chick’s metabolic maintenance for several days post-hatching [30]. Our study corroborates these observations as we also noted that the lysosomes present at hatching had disappeared entirely within 7 days. It is believed that the growth of the small intestine of birds after hatching occurs without the formation of additional villi [31]. This indicates that the villi that exist at hatching only change in size over time [31]. With age, the increase in villus size is limited. This is compensated for by increases in the microvillus length that continue with age thus enlarging the mucosal surface area [32]. Bohórquez et al. (2011) have reported a similar tendency in the growth of microvillus length of the turkey’s intestine from 1.90 μm at d 1 to 3.11 μm at 12 d post hatching [33]. In our study, this phenomenon was observed only in LD chicken between day 35 and day 63. The change in the microvilli length was limited when the increase in the villi height was permanent i.e. until day 35 in both chicken lines. Where the increase in the microvilli was obvious when the increase in the villi height was limited i.e. between day 35 and 63 in the LD chicken.

Tight junctions form a selective permeability barrier across the epithelial cell layer that are crucial for normal epithelial functions. The barrier functions of tight junctions are largely a consequence of their complex molecular composition [34, 35]. In our study of hatchlings through to 35 d or 63 d for Ross 308 and LD chickens respectively, the junctional complexes were fully developed throughout the entire study. This suggest that the intestine of the LD birds have the same barrier function on the level of the junctional complex compared to those observed in Ross chicken.

## Conclusion

The gastrointestinal tract of the LD birds was not affected by the breeding and selection criteria used in the development of this dual purpose chicken line. At gross anatomical, histological and ultrastructural levels the gastrointestinal tract of the LD birds grew proportionately to the increase in body weight without any abnormalities or deformations being observed. However, there were several anatomical differences between the LD birds and the more rapidly growing birds Ross 308 birds that may contribute to the slower growth rate of LD birds. We suggest that LD chickens have a lower nutrient absorption capacity due to their shorter intestine and smaller intestinal mucosal surface area that result in a slower body growth rate than found in Ross chickens. Moreover, the earlier establishment of the time of a balance between the growth of gastrointestinal tract segments and the overall increase in body weight in Ross chickens compared to LD chickens is noteworthy. The earlier balance time-point in the Ross birds is possibly an indicator of their better growth and performance. Thus anatomical characteristics of the gastrointestinal tract such as the time of body weight-organ balance, intestinal length and intestinal mucosal surface area could be considered as criteria for the ongoing selection of the LD birds to improve their performance and to optimize feeding strategies. However, the interactions between feeding regimens to gizzard and intestine development needs further investigation.

## Supporting information

S1 Table Mean and standard error of the mean (SEM) of body weight, mass and normalized mass of the glandular stomach and gizzard in Ross and LD chickens. BW: body weight; LD: Lohmann Dual; Ross: Ross 308; n: animal number.
(DOCX)

S2 Table Mean and standard error of the mean (SEM) of body weight and entire intestinal length, mass, normalized mass and relative length in LD and Ross chickens. BW: body weight; LD: Lohmann Dual; Ross: Ross 308; n: animal number.
(DOCX)
