# Supplementary material for: Comparison of the gastrointestinal tract of a dual-purpose to a broiler chicken line: A qualitative and quantitative macroscopic and microscopic study
Source: PLoS One. 2018 Oct 19;13(10):e0204921. doi: 10.1371/journal.pone.0204921 (PMC6195275; doi:10.1371/journal.pone.0204921)
Supplement: S1 Table — BW: body weight; LD: Lohmann Dual; Ross: Ross 308; n: animal number. (DOCX) [file pone.0204921.s001.docx]

**Supporting information**

**S1 Table**. **Mean and standard error of the mean (SEM) of body weight, mass and normalized mass of the glandular stomach and gizzard in Ross and LD chickens.**

| **Age (days)** | **Line (n)** | **Body weight (g)** | | **Glandular stomach (g)** | | **Normalized mass of the glandular stomach (g per 100g BW)** | | **Gizzard (g)** | | **Normalized mass of the gizzard (g per 100g BW)** | |
| --- | --- | --- | --- | --- | --- | --- | --- | --- | --- | --- | --- |
|  |  | **Mean** | **SEM** | **Mean** | **SEM** | **Mean** | **SEM** | **Mean** | **SEM** | **Mean** | **SEM** |
| **1** | **Ross (6)** | 52.26 | 0.94 | 0.52 | 0.03 | 1.00 | 0.067 | 2.68 | 0.06 | 5.13 | 0.12 |
|  | **LD (6)** | 42.45 | 1.25 | 0.46 | 0.04 | 1.09 | 0.087 | 2.41 | 0.12 | 5.68 | 0.21 |
| **7** | **Ross (6)** | 169.47 | 7.92 | 1.64 | 0.07 | 0.97 | 0.026 | 6.20 | 0.19 | 3.69 | 0.18 |
|  | **LD** | 101.20 | 3.32 | 1.07 | 0.05 | 1.07 | 0.062 | 4.97 | 0.09 | 4.94 | 0.23 |
| **14** | **Ross (6)** | 435.35 | 11.44 | 2.86 | 0.11 | 0.66 | 0.024 | 12.64 | 0.75 | 2.91 | 0.18 |
|  | **LD (6)** | 224.77 | 5.31 | 1.70 | 0.05 | 0.76 | 0.024 | 8.16 | 0.26 | 3.64 | 0.13 |
| **19** | **Ross (6)** | 640.73 | 45.22 | 3.18 | 0.09 | 0.51 | 0.035 | 13.83 | 0.90 | 2.17 | 0.07 |
| **21** | **Ross (6)** | 746.58 | 23.81 | 3.74 | 0.15 | 0.50 | 0.009 | 16.06 | 1.45 | 2.15 | 0.18 |
|  | **LD (6)** | 329.17 | 18.80 | 2.06 | 0.16 | 0.62 | 0.018 | 11.56 | 0.37 | 3.54 | 0.11 |
| **25** | **Ross (6)** | 1191.67 | 37.78 | 4.64 | 0.20 | 0.39 | 0.016 | 21.14 | 0.47 | 1.78 | 0.04 |
| **28** | **Ross (6)** | 1221.00 | 44.50 | 5.40 | 0.21 | 0.44 | 0.022 | 24.22 | 1.06 | 1.98 | 0.03 |
|  | **LD (6)** | 575.33 | 25.54 | 3.18 | 0.23 | 0.55 | 0.025 | 16.15 | 0.41 | 2.83 | 0.12 |
| **32** | **Ross (6)** | 1677.83 | 70.52 | 6.68 | 0.41 | 0.40 | 0.014 | 29.41 | 1.23 | 1.76 | 0.08 |
|  | **LD (6)** | 754.17 | 38.30 | 3.95 | 0.27 | 0.53 | 0.032 | 19.99 | 1.27 | 2.69 | 0.22 |
| **35** | **Ross (6)** | 2013.17 | 58.26 | 7.13 | 0.26 | 0.36 | 0.017 | 33.90 | 1.92 | 1.69 | 0.09 |
|  | **LD (6)** | 791.67 | 23.85 | 3.69 | 0.25 | 0.47 | 0.028 | 22.86 | 1.29 | 2.90 | 0.19 |
| **42** | **LD (6)** | 1130.50 | 24.15 | 4.44 | 0.11 | 0.39 | 0.013 | 25.26 | 0.84 | 2.24 | 0.08 |
| **49** | **LD (6)** | 1522.50 | 46.02 | 6.08 | 0.45 | 0.40 | 0.028 | 27.78 | 1.54 | 1.84 | 0.14 |
| **56** | **LD (6)** | 1817.33 | 54.79 | 6.22 | 0.19 | 0.34 | 0.005 | 31.45 | 1.82 | 1.73 | 0.10 |
| **63** | **LD (6)** | 2011.83 | 74.66 | 7.65 | 0.46 | 0.38 | 0.020 | 36.51 | 1.74 | 1.82 | 0.09 |

BW: body weight; LD: Lohmann Dual; Ross: Ross 308; n: animal number.
