# Supplementary material for: Comparison of the gastrointestinal tract of a dual-purpose to a broiler chicken line: A qualitative and quantitative macroscopic and microscopic study
Source: PLoS One. 2018 Oct 19;13(10):e0204921. doi: 10.1371/journal.pone.0204921 (PMC6195275; doi:10.1371/journal.pone.0204921)
Supplement: S2 Table — BW: body weight; LD: Lohmann Dual; Ross: Ross 308; n: animal number. (DOCX) [file pone.0204921.s002.docx]

**S2 Table**. **Mean and standard error of the mean (SEM) of body weight and entire intestinal length, mass, normalized mass and relative length in LD and Ross chickens.**

| **Age (days)** | **Line (n)** | **Body weight (g)** | | **Entire Intestine** | | | | | | | | | |
| --- | --- | --- | --- | --- | --- | --- | --- | --- | --- | --- | --- | --- | --- |
|  |  |  |  | **Length (cm)** | | **Mass (g)** | | **Normalized mass (g per 100g BW)** | | **Relative length (cm/100g BW)** | | **Intestinal density (g/cm)** | |
|  |  | **Mean** | **SEM** | **Mean** | **SEM** | **Mean** | **SEM** | **Mean** | **SEM** | **Mean** | **SEM** | **Mean** | **SEM** |
| **1** | **Ross (6)** | 52.26 | 0.94 | 61.83 | 1.08 | 2.67 | 0.10 | 5.11 | 0.17 | 118.48 | 2.57 | 0.04 | 0.001 |
|  | **LD (6)** | 42.45 | 1.25 | 50.87 | 1.55 | 2.14 | 0.05 | 5.08 | 0.26 | 120.21 | 4.32 | 0.04 | 0.001 |
| **7** | **Ross (6)** | 169.47 | 7.92 | 104.42 | 2.46 | 10.30 | 0.72 | 6.12 | 0.41 | 62.07 | 2.17 | 0.10 | 0.007 |
|  | **LD (6)** | 101.20 | 3.32 | 86.45 | 2.27 | 7.59 | 0.39 | 7.48 | 0.17 | 85.72 | 2.63 | 0.09 | 0.003 |
| **14** | **Ross (6)** | 435.35 | 11.44 | 132.75 | 4.06 | 22.20 | 0.73 | 5.11 | 0.17 | 30.65 | 1.48 | 0.17 | 0.009 |
|  | **LD (6)** | 224.77 | 5.31 | 102.15 | 2.16 | 11.67 | 0.46 | 5.19 | 0.14 | 45.62 | 1.72 | 0.11 | 0.004 |
| **19** | **Ross (6)** | 640.73 | 45.22 | 145.72 | 3.85 | 29.25 | 2.37 | 4.58 | 0.24 | 23.13 | 1.19 | 0.20 | 0.014 |
| **21** | **Ross (6)** | 746.58 | 23.81 | 160.53 | 4.38 | 35.19 | 1.47 | 4.71 | 0.11 | 21.57 | 0.63 | 0.22 | 0.010 |
|  | **LD (6)** | 329.17 | 18.80 | 114.98 | 2.00 | 17.44 | 1.13 | 5.29 | 0.10 | 35.41 | 1.78 | 0.15 | 0.009 |
| **25** | **Ross (6)** | 1191.67 | 37.78 | 189.03 | 3.65 | 51.73 | 1.83 | 4.34 | 0.07 | 15.92 | 0.45 | 0.27 | 0.009 |
| **28** | **Ross (6)** | 1221.00 | 44.50 | 192.53 | 5.76 | 52.01 | 2.25 | 4.26 | 0.11 | 15.85 | 0.63 | 0.27 | 0.008 |
|  | **LD (6)** | 575.33 | 25.54 | 140.98 | 3.65 | 27.81 | 1.03 | 4.84 | 0.09 | 24.67 | 0.96 | 0.20 | 0.006 |
| **32** | **Ross (6)** | 1677.83 | 70.52 | 206.12 | 4.23 | 69.56 | 2.07 | 4.16 | 0.13 | 12.36 | 0.44 | 0.34 | 0.006 |
|  | **LD (6)** | 754.17 | 38.30 | 155.60 | 5.67 | 38.18 | 1.08 | 5.10 | 0.16 | 20.82 | 0.99 | 0.25 | 0.010 |
| **35** | **Ross (6)** | 2013.17 | 58.26 | 217.28 | 2.40 | 86.71 | 2.68 | 4.31 | 0.11 | 10.84 | 0.37 | 0.40 | 0.016 |
|  | **LD (6)** | 791.67 | 23.85 | 152.23 | 6.10 | 39.12 | 2.00 | 4.95 | 0.22 | 19.31 | 1.00 | 0.26 | 0.009 |
| **42** | **LD (6)** | 1130.50 | 24.15 | 168.45 | 4.70 | 47.66 | 2.04 | 4.21 | 0.13 | 14.92 | 0.45 | 0.28 | 0.011 |
| **49** | **LD (6)** | 1522.50 | 46.02 | 174.04 | 4.40 | 55.67 | 1.72 | 3.66 | 0.06 | 11.45 | 0.21 | 0.32 | 0.004 |
| **56** | **LD (6)** | 1817.33 | 54.79 | 169.87 | 3.22 | 65.95 | 2.98 | 3.63 | 0.15 | 9.40 | 0.38 | 0.39 | 0.017 |
| **63** | **LD (6)** | 2011.83 | 74.66 | 182.95 | 4.99 | 64.61 | 3.32 | 3.20 | 0.05 | 9.12 | 0.21 | 0.35 | 0.013 |

BW: body weight; LD: Lohmann Dual; Ross: Ross 308; n: animal number.
